# Intersections of the arts and art therapies in the humanization of care in hospitals: Experiences from the music therapy service of the University Hospital Fundación Santa Fe de Bogotá, Colombia

**DOI:** 10.3389/fpubh.2022.1020116

**Published:** 2022-12-02

**Authors:** Mark Ettenberger, Nayibe Paola Calderón Cifuentes

**Affiliations:** ^1^Music Therapy Service, University Hospital Fundación Santa Fe de Bogotá, Bogotá, Colombia; ^2^SONO – Centro de Musicoterapia, Bogotá, Colombia; ^3^Department of Social Management, University Hospital Fundación Santa Fe de Bogotá, Bogotá, Colombia

**Keywords:** music therapy, humanization of care, hospitals, person-centered care, art, therapy

## Abstract

Humanization of care is becoming an increasingly important aspect in providing high-quality health services and the arts are more and more implemented to support and foster humanization and person-centered care efforts. Musical experiences are one of the most frequently encountered art forms in medical settings. Music therapy as a healthcare profession has a decades-long tradition in hospitals, both in inpatient and outpatient areas. However, while studies regarding the effectiveness of music therapy are on the forefront of clinical research, little attention has been paid to the profession's inherent opportunities to assist the hospitals' strategies in terms of humanization of care. Yet, the musical experiences in music therapy are especially versatile in supporting healthcare users from a holistic perspective, contributing to a more compassionate, personalized, and humanized environment. In this article, the basic pillars of humanized and person-centered care will be outlined, followed by examples of seven intersections in which the music therapy service of the University Hospital Fundación Santa Fe de Bogotá aligns with its Humanized and Compassionate Care Model. The aim of this article is to stimulate the discussion on music therapy not only as a profession that provides safe and effective treatment, but also as a therapeutic art experience that can add value for hospitals on their path toward a more humanized care culture.

## Introduction

Humanization of care is a set of relational, organizational, and structural strategies and values leading toward a lived culture of care that puts the uniqueness of patients,^1^ their needs, and potentials, at its center (1). Thus, the path toward humanized care is always systemic, including communicational, personal, and emotional skills of healthcare professionals (2–4), hospital infrastructure (5–7), and adequate resources for staff and technology (7, 8).

Inherent to a humanized care model are the concepts of patient-centered and person-centered care. Patient-centered care is defined as care, in which “…an individual's specific health needs and desired health outcomes are the driving force behind all health care decisions and quality measurements. Patients are partners with their health care providers, and providers treat patients not only from a clinical perspective, but also from an emotional, mental, spiritual, social, and financial perspective” [(9), np.]. Thus, the individualization of treatment, empowerment, dignity and respect, shared decision-making, and transparent communication, are all cornerstones of patient-centered care (10). Person-centered care shares those values (11) but transcends patient-centered care in relation to its goal: “The goal of patient-centered care is a functional life for the patient while the goal of person-centered care is a meaningful life for the person” [(12), p. 13]. In other words, person-centered care not only seeks to know what helps a patient, but also what matters to a patient. This shift of focus leads to a more holistic approach to healthcare, making it necessary to consider a person's life trajectory, expectations of health outcomes, and how individuals understand, process, and respond to illness, stress, and trauma. The unique interplay which situates each and everyone in a specific historical moment, geographical region and cultural community becomes thus the main incentive in the process of humanized care. This provides an opportunity to think beyond established ideas of what healthcare services should look like and integrate *in situ* resources and technologies for health and healing, including the therapeutic applications of the arts.

Consequently, art programs in hospitals often resonate with the ideas of humanization and national programs for arts in health exist for example in Australia (13), the United States (14) or several European countries (15). Arts in health refers to “…a diverse, multidisciplinary field dedicated to humanizing the healthcare experience by connecting people with the power of the arts. This rapidly growing field integrates the arts, including literary, performing, and visual arts and design, into a wide variety of healthcare settings for therapeutic, educational, and recreational purposes” [(16), p. 3]. Reviews on the effectiveness of various artforms in medical settings underline the physiological and psychological benefits for users, staff, and caregivers (17, 18). Recently, one of the largest reviews on this topic was commissioned by the WHO, covering more than 3,000 studies on arts and health (15). The authors conclude that “…the arts can provide a holistic lens to view conditions that are often treated primarily as physical; this approach fits with current trends in health toward giving parity of esteem to mental health and also toward situating health problems within their social and community context” [(15), p. 53]. Despite the critique the WHO report has received questioning the robustness of the evidence (19, 20), this conclusion aligns well with the previously outlined concepts of humanized and person-centered care.

While many different art experiences have found their way into healthcare settings, music is certainly one of most frequent ones. Music has been part of health practices for thousands of years (21, 22) and publications regarding the role of music and musicians in contemporary hospitals date back to the 19th century (23). With the establishment of music therapy as a profession in the late 1940s and early 1950s, music has been increasingly used by trained music therapists (i.e., music therapy), or other healthcare professionals (i.e., music medicine). The differences between music therapy and music medicine have been clearly established. Music therapy is an individually tailored and relationship-based therapy, in which the music and the therapeutic relationship are used as a mechanism of change and as a vehicle to modulate attention, emotion, cognition, behavior, and communication (24). Music medicine on the other hand involves mostly the listening of pre-recorded music provided by other healthcare professionals and usually does not involve a therapeutic relationship (24). Lately, also other music practitioners form part of the hospital landscape, such as healthcare musicians or hospital musicians. While overlaps to music therapy and music medicine might be given in specific situations, healthcare musicians have different professional orientations, stances, and goals (25).

Over the last decades, clinical research in music therapy and music medicine have expanded exponentially, providing evidence for their benefits in a myriad of populations, such as preterm infants (26, 27), cancer patients (28), or burn patients (29, 30), for example. Also, topics of transversal importance, such as pain (31), stress (32), insomnia (33), or end-of-life care (34, 35), have been investigated. More recently, potential mechanisms, moderators, and mediators of music-based interventions are beginning to be examined, demonstrating a shift of the field toward “second-generation research” (32, 36). Thus, clinical practice in music therapy is clearly evidence-based and goal-oriented; yet it is both science and art, both treatment and experience. Most research focuses however on the “science-treatment” aspects of music therapy. This is reasonable and important, since decisions regarding best available care for patients need to be based on the pillars of safety, effectiveness, and ethics. But this also means that the “art-experience” aspect of music therapy is often regarded as secondary, although it is those characteristics that are most often articulated in terms of humanization of care.

In this article we try to highlight the inherent—and often understated—potential of music therapy services to not only provide safe and effective treatment, but also to help hospitals reach their goals in generating a more compassionate and personalized care culture. It is hoped that this will lead to a more holistic view of music therapy services in medical contexts and potentiate what is at the heart of the music therapy profession—the integration of art and science.

## Music, music therapy, and humanization of care

The idea that music can be helpful in terms of humanization of care is not new. Already more than 20 years ago, nursing researchers advocated for non-pharmacological and adjunctive interventions to humanize intensive care, including music (37). In 2004, Bergold and Sobral described several aspects of music therapy in terms of humanization, such as providing comfort, generating familiarity and empathy, helping to express feelings, and break hierarchies and resistances (38). Other early articles on the topic mention music therapy as an opportunity to reduce psychological distress, provide an integral view of the person, collaborate with other healthcare professionals, and enrich the hospital environment (39, 40).

Over the last years, an increasing number of studies have been published trying to connect the clinical effects of music therapy with the ideas of humanization. Many of these articles part from the idea of a holistic approach to healthcare and point out that music therapy can help in taking into account the biopsychosocial aspects of hospital users (6, 41). Examples hereof are Zanini et al.'s work of music therapy with hypertensive patients (41) or Pimentel's discussion of the convergence of music therapy and the National Humanization Policy in Brazil (42). In end-of-life care, Tao (43) described the importance of music therapy in establishing a sense of personhood, strengthening relationships between patients, families, and staff, generating spiritual meaning, and in prioritizing the person's identity as a human being. Studies on music medicine describe how music can provide integrative care as an interactive, participative, and recreational way to engage staff, users, and families (44, 45). However, in most articles, humanization of care as a concept with specific dimensions is often poorly described, and a more systematic description of the potential interplay between music therapy and person-centered and humanized care is lacking.

In the following section, the basic pillars of the Humanized and Compassionate Care Model of the University Hospital Fundación Santa Fe de Bogotá (FSFB) will be described and examples of seven intersections with clinical practice of the music therapy service at the FSFB will be outlined.

## The humanized and compassionate care model at the University Hospital Fundación Santa Fe de Bogotá in Colombia

The University Hospital FSFB forms part of the wider healthcare network of the foundation and as a private institution with social goals, the hospital opened in 1983. Since then, the FSFB has become one of the most renown hospitals in Colombia and a hub for innovation and development of the health sector in the country. The hospital has currently 329 beds and in 2021, the 2,506 staff members provided care for 16,431 hospitalizations. Part of the hospital community form both national and international patients and caregivers, and since the FSFB is a university teaching hospital, also students and residents rotate at the different departments. The central mission of the FSFB is the provision of services with the highest quality and outstanding patient safety management. On these pillars, the hospital's Humanized and Compassionate Care Model is built. In this model, quality and safety of care are combined with empathy and compassion, emphasizing the respect for autonomy, a needs-based and culture-sensitive communication between staff, patients and families, and the rights of intimacy and privacy of healthcare users. Thus, person-centered care is understood as a path that contributes to better health. As part of the implementation of the Humanized and Compassionate Care Model, the hospital has developed different strategies and programs for patients, families, staff, and the hospital community in general. This has made it possible to transform the hospital environment into a space where people can feel comfortable, calm, motivated and empowered for their self-care.

## *Arte-Sano*: The craft of art and health

One of the strategies that stands out at the FSFB is *Arte-Sano*, which resulted from the need to generate art-based recreational, educational, and therapeutic environments for service users and caregivers, leading to a boost of innovation in the artistic and cultural sector of the hospital. *Arte-Sano* is a play on words in Spanish, with *arte* (art), *sano* (healthy) and *artesano* (craftsman), derived from *artesan*í*a* (craft). *Arte-Sano* forms part of the Department of Social Management and seeks to create a balance between physiological, psychological, and social needs through the arts in both inpatient and outpatient settings, seeking a warmer, more humanized, and personalized care experience. Under the umbrella of *Arte-Sano*, a variety of art experiences are offered for the hospital community, such as art exhibitions, art workshops, storytelling, among others.

In terms of scope and emphasis, among the most important programs of *Arte-Sano* is “Hospital full of music”, which started in 2015. Under this program, music is used as the main element of wellbeing, helping in the therapeutic processes of patients and caregivers, focusing on emotion regulation, motivation, and joy through a variety of musical experiences. Within the program, music therapy, professional music, and environmental music have been implemented each on distinct levels within the care process. Environmental music is provided in waiting areas, the outpatient service, and the emergency service, and consists of carefully selected pre-recorded music with the aim to generate comfort in healthcare users, caregivers, and staff. Hospital musicians provide live music in high-impact areas such as post-surgery areas, the renal unit or in the chemotherapy area for example. Professional musicians, together with patients, visitors, and collaborators, offer scheduled concerts in the entrance hall open to the entire hospital community.

## The music therapy service at the FSFB

The music therapy service forms part of the program “Hospital full of music” and started in early 2017 with a small-scale pilot project at the Neonatal Intensive Care Unit (NICU). Over the past 5 years, the program grew to an integral service at the Neonatal ICU, Pediatric ICU, Adults ICU and Oncology including chemotherapy, hospice, and general hospitalization. In its scope and level of innovation unique to the hospital landscape in Colombia, the team consists currently of five certified music therapists, providing musical care for over 1,500 patients and families through more than 3,000 music therapy sessions per year. The music therapy team is funded by the hospital itself (contracted by the Department of Services) and currently provides 87 h of music therapy per week. All referrals are done by the medical staff, the mental health team, or the palliative care team. Besides one-to-one sessions, group sessions are carried out weekly at the chemotherapy unit and at the pediatric inpatient unit. Environmental Music Therapy (EMT) is provided on demand or in times of COVID-19 peaks in restricted areas. Additionally, monthly music self-care workshops are led by the music therapy team for the hospital community, such as parents of hospitalized infants, oncology patients and their caregivers, people with Alzheimer, or users and staff of the hydrocephalus care unit, among others. Thus, while being a fully integrated member of the interdisciplinary healthcare team and thus clearly articulated in its “science-treatment” aspect, the “art-experience” aspect of the music therapy service is explicitly aligned with the institutional goals to create a lived culture of humanized and person-centered care.

Following the nine key elements of person-centered care as described in Håkansson Eklund et al. (12) (empathy, respect, engagement, relationship, communication, shared decision-making, holistic focus, individualized focus, coordinated care) and the three areas of humanized care as described in Busch et al. (1) (relational, organizational, structural), we have identified seven main intersections, in which daily clinical practice of the music therapy service at the FSFB is linked to the concepts of humanized and person-centered care. While the way these intersections manifest themselves are specific to the context of the FSFB, many hospital-based music therapy services might align their work along similar thoughts. Thus, some of these intersections could be applicable to other contexts too.

### Individualization of care experiences and shared decision-making

Individualization of treatment, considering each patient's needs and resources, is best practice in person-centered care, but also in music therapy. While music therapists use a variety of methods and techniques according to clinical goals, how users want to be engaged in music is often negotiated and based on their preferences. The idea of a collaborative approach to healthcare, in which decisions regarding treatment are taken together with patients, families, and music therapists, is firmly embedded in Colombian hospitals (46). Music with personal, social, and cultural meaning for patients is at the heart of many music therapy interventions at the FSFB and is a way to honor a patient's biography and history. In this way, the music in music therapy is based on, but also transcends clinical goals. Preferred music can help to ease pain or modulate mood and attention of a patient, but it is also testimony of the person's life, shared between the music therapist, patient, family, and staff. Music with personal meaning elicits emotions, sensations, and memories, which intrinsically connect all biopsychosocial domains of health and wellbeing, a key feature of person-centered care (12).

### Culture-sensitive practice

A true humanized care approach is always sensitive to culture and based on the respect for traditions, customs, and values. Music is an excellent medium through which a patient's needs and expectations can be situated within his or her social and cultural community. Culture-centered or culture-specific practice has been relevant for years within the community music therapy landscape (47, 48), and has slowly found its way also into the field of medical music therapy (49, 50). “If you do not respect the culture of another person then you do not respect the person. Culture enables and restricts human life and development and Culture-Centered Music Therapy facilitates constructive and critical re-examination of practice, theory, and research in this light. More than a stimulus influencing human behavior, culture is considered a resource for action and an integral element in human interaction” [(48), p. 538]. This quote outlines some of the main thoughts of culture-centered practice, but also hints at the challenges when taking culture seriously. The FSFB is one of the leading university hospitals in Colombia and a multicultural place. Colombia itself is incredibly rich in terms of cultural diversity and patients and families may come from such different regions as the Amazon, the Caribbean Coast, or the Andean Highlands for example. Besides, international patients also form part of the hospital community. Thus, the music in music therapy needs not only be meaningful, but also culturally appropriate. Examples from the clinical service at the FSFB include working with orthodox Jewish mothers in the Neonatal ICU, who usually would not sing in public spaces or in front of men, including music therapists. Accompanying spoken prayers by live music can thus be more acceptable for those mothers and may be a way of respecting cultural norms, provide the newborn with the important experience of hearing his mother's voice, and help the mother achieve a sense of self-efficacy and empowerment. In other situations, being respectful of culture might simply mean not to use any music at all, or to consult first with specific members of a patient's social network, as it is the case with hospitalized members of indigenous communities for example. While the choices of musical instruments also form part of a culturally reflective practice, many indigenous instruments made from organic material (seeds, untreated wood, animal skins, etc.) can unfortunately not be used in intensive care due to biosafety reasons. Specific instruments such as rain sticks, or ocean drums are either used in a plastic version or wrapped in synthetic leather that can be cleaned accordingly (51).

At the same time as music therapists try to be aware and respectful of cultural norms and expectations regarding music and health, the music therapy service at the FSFB is in itself a driving force of culture. As music therapy is a relatively new profession in Colombia and not commonly found in hospital settings, establishing a “culture of music therapy”, in which the therapeutic use of music forms part of the culture of care in even the most critical areas such as in intensive care, transforms how music as an artform and therapy is perceived and understood within the hospital system.

### Integration of families and caregivers

Every patient forms part of multiple social networks and communities. The closest social networks (families, friends) are usually also those who accompany a patient if a hospitalization is required. Thus, families and caregivers are important allies in person-centered and humanized care but might also need care on their own. Secondary trauma, worries, or increased anxiety levels can negatively affect mental health and wellbeing of caregivers of critical care patients (52–54), which in turn might negatively influence the support a patient requires during hospitalization. At the FSFB, families and caregivers frequently have an opportunity to actively take part in music therapy. Whether through participating in music assisted relaxation experiences, through singing songs that reflect their history with the patient, or through participative therapeutic songwriting; there are many ways in which family members or caregivers can get a sense of empowerment through musical co-caring and simultaneously work on their own wellbeing. In specific contexts, for example in the Neonatal ICU, parents and families are equally important participants in music therapy as their infants. In fact, many times the attending neonatologist, the social worker, or the mental health care team might refer an infant to music therapy for working on parental mental health or for fostering the parent-infant relationship. Family-centered music therapy is firmly integrated in the NICU of the FSFB and other Colombian hospitals (46). Integrating both mothers and fathers to both clinical music therapy research in the NICU has been pioneered in Colombia, resulting in improved bonding, less anxiety in mothers, and a better weight gain for the babies (55). Singing songs with personal meaning or writing welcome songs for their babies are just a few of the music therapy experiences that can help in the integration of parents to the care process (56). Music therapy self-care groups for fathers and mothers of a NICU infant also take place regularly. In these group sessions, participants can actively foster their self-care and reflect together with other parents on their journey in the NICU (57).

### Knowledge transference

Patient and caregiver education is key to humanized care (4). Providing tips and guidance on the safe and purposeful use of music during and after hospitalization forms part of the educational support that music therapy services can provide for patients, caregivers, and staff. In 2021, the music therapy service at the FSFB provided eight workshops on music and self-care for the hospital community, benefitting more than 200 participants. Short articles about specific topics on music and wellbeing are written bimonthly by the music therapy team and published at *Arte-Sano's* own hospital journal. In the areas in which environmental music is played *via* speakers, after every couple of songs, a “healthy tip on music” is played back, which has been written by the music therapy team and has then been recorded by a professional radio host. During the peaks of COVID-19 and the subsequent restrictions for one-to-one sessions at certain areas of the hospital, the music therapy team elaborated music-assisted relaxation recordings, which were sent to nurses and staff for their own self-care and for the use of patients in the COVID area. Additionally, flyers and leaflets regarding how to use music during and after hospitalization have been created for children, adults, and oncology patients. In this way, patients, caregivers, and staff can be empowered for their own health and wellbeing, according to their resources, abilities, preferences, and cultural context.

### Modulating the hospital sound environment

Music therapists regularly modulate the music according to environmental goals. Creating a calm, comfortable and safe hospital environment is key for trauma-informed care (58), but also an important factor in terms of humanization (7, 59, 60). Environmental Music Therapy (EMT) is described as “…a human-centered, trauma informed strategy that encompasses a process using the metaphoric and associative properties of live music that seeks to modulate patients' and staff's perception of the hospital milieu as hostile” [(61), p. 130]. Several studies have shown that EMT can reduce stress and improve noise perception in hospital areas (61, 62). At the FSFB, EMT is mostly applied in specific areas inside the hospital, for example at the chemotherapy unit, but also during times in which individual or group sessions might be restricted due to a COVID-19 peak. As also hospital or healthcare musicians apply environmental live music at the FSFB, the boundaries between environmental music and EMT can get blurred. However, healthcare musicians perceive their work differently compared to music therapists and base their interventions according to distinct goals and frameworks (25, 63). Music therapists create music during EMT considering the current needs of patients and families (e.g., anxiety, stress, boredom, etc.), the characteristics of the space (open, closed, transit, etc.) and the existing sound environment (alarms, voices, calls, etc.). While written musical pieces may form part of the EMT repertoire, the music is often improvised and pieces—if used—are interwoven in musical improvisation. Also, music therapists closely observe the environment, and song choices or the use of musical parameters are based on a previous assessment. Finally, the music is used according to clinical goals and not to the music itself. Hence, EMT can be another effective way for music therapists to improve user and staff wellbeing and contribute to a more humanized hospital environment.

### End-of-life care

End-of-life care is certainly one of the areas in which humanization of care is most firmly established. The transition from restorative to palliative care is a frequent referral criterion for music therapists in hospital settings and many studies indicate that music-based interventions can improve quality of life, reduce pain, and provide comfort during end-of-life care (64–66). At the FSFB, the music therapy service is regularly asked to accompany patients, families, and caregivers during end-of-life care. This is not only the case for oncology patients, but also in the ICUs, where the elaboration of rituals, memory-making, and family participation are the cornerstones of the music therapy process (67). Furthermore, the music therapy service accompanies weddings, baptize rituals or other religious ceremonies for palliative care patients inside the hospital. Recently, the team has been integrated to the perinatal grief committee and music therapy groups for parents and families in grief are beginning to be implemented.

### Coordinated care and teamwork

Coordinated care is one of the key elements of humanized care (1). While multidisciplinarity is common throughout the FSFB, music therapy is especially versatile to additionally work on an interdisciplinary level. Accompanying other therapy services (e.g., physiotherapy, speech and language therapy, occupational therapy, respiratory therapy, etc.) is common clinical practice for many hospital-based music therapists. Procedural support is regularly provided in a shared space with medical and nursing staff. A recent review indicates that music therapy during procedural support could help patients reduce their pain and anxiety levels (68). In the Neonatal ICU, music therapy has been pioneered in guiding parents to use music during procedural support for their babies (69, 70). And in pediatric care, a recent study suggests that a combination of child life and music therapy intervention can help reduce stress in children during intravenous placement (71). Often, music therapists take part in medical rounds, discuss referrals with medical staff, mental health teams, or palliative care teams, and work together with patients, families, and caregivers. Therefore, music therapy both coordinates with and extends a multidisciplinary approach to healthcare and creates a space for musical co-caring and co-occupation (72).

## Measuring impact

While clinical research is an important part of making visible the benefits of music therapy services for patients, caregivers, and the hospital environment, their impact in terms of humanization or person-centered care has not been measured yet. Formal outcome measures regarding humanized and person-centered care have been proposed by different disciplines, such as nursing science (73, 74) or psychology (3) for example. Patient experience can also be a useful barometer to evaluate the impact of humanized care efforts. Patient experience is conceptualized “…both as patients' experiences of care and as feedback received from patients about those experiences” [(75), p. 236]. A positive patient experience is important for hospitals, since it is not only a quality-of-care measure, but also linked to better health outcomes (76, 77). In the case of the music therapy service of the FSFB, patient experience is measured *via* questionnaires that are handed out to patients or families/caregivers at the end of the music therapy process. Each questionnaire includes questions according to four categories (see the full questionnaire in Appendix 1):

- Patient/caregiver satisfaction
- Effectiveness
- Humanization
- User fidelity

Since the start of the service, 531 questionnaires have been handed out across the different units, resulting in 98.4% of patients and families expressing being very satisfied with the music therapy service, 97.1% stating that music therapy helped a lot in their recovery (average as either answered by patients or family members), 100% believing that music therapy helps making the care at the FSFB more humane, and 100% recommending the FSFB to family members or friends for having a music therapy service. However, since the questionnaires are usually handed out and analyzed by the music therapy team itself, there might be bias involved. To mitigate this bias, the methodology currently transits to using a QR code for the questionnaires, which patients and family members can scan and then fill out online. Other more detailed service evaluation tools have been proposed by Tsiris et al. (78) for example, which can either be filled out by patients, staff, or music therapists. At the FSFB, it is expected that patients themselves fill out the questionnaires, with exception of young infants, for whom the questionnaire is filled out by their caregivers. This is a challenge since the health status of intensive care patients can fluctuate considerably (and thus their ability to answer questions). Moreover, the music therapy service concentrates its resources on the ICU, and follow-up visits of patients after ICU discharge is not provided on a regular basis. Besides the issue of service and impact evaluation, also cost-effectiveness of music-based interventions are highly relevant, but certainly warrants further investigation (17).

## Conclusions

The arts including music can bring multiple benefits for hospitals and can support their goals regarding humanization of care. Music therapy as a healthcare profession has a long-lasting tradition of clinical practice and research in medical settings. Besides providing successful and safe treatment for patients and families, music therapy services are particularly versatile in working from a holistic perspective, connecting all important domains of health: physiological, emotional, mental, social, and spiritual. Since the integration of art and science is at the heart of the profession, humanization of care can be a suitable framework for music therapists to equally value and foster both the “science-treatment” and “art-experience” aspects their work. Policy makers can hopefully recognize this “double potential” and help thrive not only music initiatives, but also music therapy services in hospitals. It is important for healthcare institutions to be aware of how, for whom and at what level music is implemented. Certainly, music therapists do not necessarily have to be present on all levels. The spectrum from clinic to community and from recreational music to music therapy is sufficiently broad for a variety of actors, who can bring in different expertise, skills, and knowledge. Music therapists can offer guidance for such processes based on current evidence and best practice. This is paramount, because as with every other healthcare intervention, music is not risk-free and models discussing the potential of harm in music therapy are finally being established within the field (79, 80). Hence, informed decisions should be made about what type of music experiences are best suited for what type of situation, contexts, and populations.

Yet, it should be stressed that concepts and theories are never free of politics, and that the notion of humanization can have different meanings in different contexts, disciplines, and historical moments (60). While a more thorough critique of the concept of humanization itself goes beyond the scope of this article, continuing efforts in this direction are undertaken from such diverse disciplines as medical history (81), development studies and sociology (82), or medical anthropology (83), just to mention a few. While at first it might be hard to argue that for example a more compassionate and empathic environment for patients and families could be worth any critique, the danger of detaching context and culture from the discourse warrants attention. After all, being empathic and compassionate can mean different things for different people at different moments. Recent studies on empathy and aggression from a music therapy perspective highlight the importance of the subjective (and thus socially and culturally embedded) worldview of the participants when it comes to experiencing empathy (84). Similarly, the impact of spiritual or religious beliefs of music therapists on their perception of empathy further adds to the complexity, indicating that divergent meanings of empathy may not only be found among healthcare users, but also among healthcare professionals (85). Thus, humanization of care might look differently according to different cultural and geographical regions, and such diversity should be appreciated. As humanization of care is essentially a process of cultural and social transformation, being reflective upon one's own stances and worldviews (from a personal, professional, and institutional perspective) is at the core of the concept.

As music therapy services continue to claim their place within hospital landscapes internationally, it is expected that such efforts eventually spill over to countries in which the profession is not yet fully recognized, as it is the case for Colombia. Humanized and person-centered care programs can be an appropriate platform for such endeavors. Hopefully this will lead not only to the establishment of new music therapy services in the country, but foremost to a culture of care that reflects what humanization truly stands for.

## Author contributions

The draft of the manuscript was written by ME and NC commented and added on previous versions of the manuscript. All authors contributed to the conception and design of the article. All authors contributed to the article writing process, read, and approved the final manuscript.

## Conflict of interest

The authors declare that the research was conducted in the absence of any commercial or financial relationships that could be construed as a potential conflict of interest. The reviewer AP declared a past collaboration with the author ME to the handling editor at the time of review.

## Publisher's note

All claims expressed in this article are solely those of the authors and do not necessarily represent those of their affiliated organizations, or those of the publisher, the editors and the reviewers. Any product that may be evaluated in this article, or claim that may be made by its manufacturer, is not guaranteed or endorsed by the publisher.

## Footnotes

^1^In this article, the authors will use the term “patient”, “healthcare user”, “person” (if clear that the primary healthcare user is meant), or similar terms, synonymously.
