# Supplementary material for: Intersections of the arts and art therapies in the humanization of care in hospitals: Experiences from the music therapy service of the University Hospital Fundación Santa Fe de Bogotá, Colombia
Source: Front Public Health. 2022 Dec 2;10:1020116. doi: 10.3389/fpubh.2022.1020116 (PMC9757166; doi:10.3389/fpubh.2022.1020116)
Supplement: Supplementary file 1 [file Data_Sheet_1.PDF]

User Experience Questionnaire – Music Therapy Service FSFB

Dear patients, family members, and caregivers,

For the music therapy team, it is very important to get your feedback to continuously improve our service and provide the best quality care. For this reason, we invite you to answer the following questions. Afterwards, please hand back this questionnaire to a music therapist or another member of the health team. Thank you very much!

1. How satisfied were you with the music therapy service?

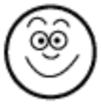 ☐ Very satisfied

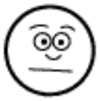 ☐ Satisfied

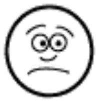 ☐ Not satisfied

2. Do you consider that the music therapy service helps to humanize the care at the Hospital Fundación Santa Fe de Bogotá?

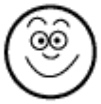 ☐ Yes, a lot

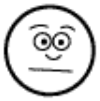 ☐ Yes, somehow

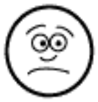 ☐ No

3. Question for family members/caregivers: Do you consider that music therapy helped in the recovery of the person hospitalized?

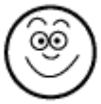 ☐ Yes, a lot

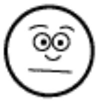 ☐ Yes, somehow

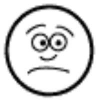 ☐ No

4. Question for the patient: Do you think music therapy helped in your recovery?

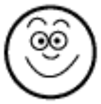 ☐ Yes, a lot

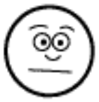 ☐ Yes, somehow

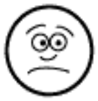 ☐ No

5. Would you recommend the FSFB to a family member or friend due to your experience in music therapy? ☐ Yes ☐ No

Below you can write your suggestions or leave us a comment about your experience in music therapy:

____

____

____

____

____
